# Cherry microbiota and metabolites with planting altitude of *Coffea arabica* in Baoshan of China

**DOI:** 10.3389/fnut.2026.1817512

**Published:** 2026-05-04

**Authors:** Xiaojing Shen, Qi Wang, Jing Gao, Wenhua Chen, Kunyi Liu, Jilai Zhang, Weiwei Jiang

**Affiliations:** 1 College of Science & College of Resources and Environment, Yunnan Agricultural University, Kunming, China; 2 School of Wuliangye Technology and Food Engineering, Yibin Vocational and Technical College, Yibin, China; 3 School of Resources and Environment, Baoshan University, Baoshan, China

**Keywords:** *coffea arabica*, flavor, metabolites, microbial diversity, planting altitude

## Abstract

The planting altitude of coffee is one of the important influencing factors on coffee quality. The effects of four planting altitudes (1,000 m, AT1; 1,200 m, AT2; 1,400 m, AT3; and 1,600 m, AT4) on the microorganism community and the related metabolites in *Coffea arabica* cherries were investigated using Illumina-based amplicon sequencing and UPLC-MS/MS technologies, respectively. At tha same time, the contents of main flavor precursor substances (caffeine, trigonelline and chlorogenic acid) were analyzed using HPLC method. The results showed that planting altitude can significant influence the microbiomes’ characteristics and endogenous metabolites of coffee. The dominant bacterial genera, *Sphingomonas* and *Pleomorphomonas* were significant differences in four planting altitudes, with the maximum relative abundance in AT3. *Cladosporium* and *Strelitziana* were the dominant fungal genera, while *Vishniacozyma* and *Setophoma* were also important in AT1, *Setophoma* and *Didymella* in AT3. 22 (AT2 vs. AT1), 68 (AT3 vs. AT1), 38 (AT3 vs. AT2), 77 (AT4 vs. AT1), 57 (AT4 vs. AT2), and 116 (AT4 vs. AT3) differentially changed metabolites were found between different planting altitudes, respectively. Moreover, caffeine, trigonelline and chlorogenic acid content was the decreas with planting altitude increased. These changes with planting altitude indicted a potential chance to find more fermentation microorganisms.

## 1 Introduction

Agriculture plays a crucial role in poverty alleviation, food security, and economic growth, which is easily influenced by pests, weather, soil, and other complex factors (1, 2). Altitude is a critical ecological and environmental factor influencing the spatial distribution of farmland, which can significantly affect the quality of agricultural products based on its combined effect of integrating variables such as temperature, humidity, light exposure, and soil properties (3–6). For agricultural products, their chemical composition, nutrient elements, and pharmacological effects are significantly altered by altitude (7–9). Additionally, the growth of plants is also affected by altitude (10).

Coffee, the most widespread global beverage and traded commodity, is not only vital to tropical agriculture and international trade, but also provides substantial economic support and employment in more than 80 cultivation countries (11, 12). Coffee thrives in altitude regions between 600 m and 2,000 m above sea level. This unique environment, characterized by lower temperatures, generous rainfall, and permeable soil, is ideal for coffee cultivation (13). Moreover, altitude is an important influencing factor on coffee’s physical quality, flavor quality and yield (14, 15). With increasing altitudes, the aroma and flavor of coffee are enhanced and the percentage of defective beans decreases (16, 17). Simultaneously, coffee chemical components and volatile compounds are also affected significantly by planting altitude (3). In addition, the microbial community structure and pharmacological profile of coffee also changed with planting altitude (18, 19). The fatty acids and chlorogenic acid contents in coffee decreased with increasing altitude (3, 20). The contents of caffeine and trigonelline fluctuated with increasing planting altitudes (3). Compared with high altitude coffee, low altitude coffee had higher contents of volatile alcohols, while lower contents of sterols, aldehydes, and total phenolics in low altitude coffee (21). In low altitude coffee, the genera *Gluconobacter* and *Weissella* were dominant (21). Therefore, the function of planting altitude on coffee is worthy of further study. Furthermore, planting altitude influenced coffee fermentation by microorganisms.

Coffee cultivation in Yunnan Province dates back to the early 20th century. Based on the local favorable geographical location and natural conditions, Yunnan has become the largest coffee-producing region in China, accounting for more than 98% of its production area and total output (3, 22, 23) and Yunnan arabica coffee has a high reputation in the international market for its unique flavor and high quality, with a sweet aroma and aromatic acidity (24). With the development of Yunnan arabica coffee production and trade in the international market, coffee cultivation is key for coffee industry development (25). Therefore, to evaluate the potential function of planting altitude on coffee, coffee from Baoshan, Yunnan, which is one of the main coffee production regions in China, was chosen as the subject. Coffee samples were collected from the same area at altitudes ranging from 1,000 to 1,600 m to analyze the microbial variety and metabolites and assess the sensory characteristics in this paper.

## 2 Methods and materials

### 2.1 Reagents and plants

Fully ripe red *Coffea Arabica* cv. Cartim coffee cherries with uniform size, color without visible disease and mold at elevations of 1,000 m (low altitude), 1,200 m (low altitude), 1,400 m (medium altitude), and 1,600 m (high altitude) above sea level were hand-picked in March 2025, which were marked AT1, AT2, AT3, and AT4, respectively. These four coffee samples were drawn from the same farm in Xinzhai village, Lujiang town, Longyang district, Baoshan City, Yunnan Province, Southwest China (25°1′N, 98°49′E), which has an average annual temperature of 21.3 °C, 268 mm of rainfall, and 2,334 h of sunshine. In addition, three biological replicates per altitude were designed. The coffee cherry samples were stored at −80 °C for Illumina-based amplicon sequencing and UPLC-MS/MS analysis. Simultaneously, they were processed using wet processing method to obtain green coffee beans for caffeine, trigonelline and chlorogenic acid analysis. Finally, the green coffee beans were roasted for sensory analysis. Acetonitrile and methanol of high-performance liquid chromatography (HPLC) grade were supplied by Thermo Fisher Scientific (Pittsburgh, United States), while the formic acid was supplied by CNW Technologies GmbH (Düsseldorf, Germany). Trigonelline (98.0%) was purchased from Acmec Biochemical Co., Ltd. (Shanghai, China). Chlorogenic acid (98.0%) was purchased from Aladdin Biochemical Technology Co., Ltd. (Shanghai, China). Caffeine (98.0%) was prepared and tested for purity by HPLC.

### 2.2 Caffeine, trigonelline and chlorogenic acid examination

Caffeine, trigonelline and chlorogenic acid of the green coffee beans were extracted by the ultrasound-assisted extraction method. The analysis was executed by modifying the method of Hu et al. (3). 0.3 g of coffee sample powder and 50 mL of MeOH-0.1% acetic acid- water (1:1) were sonicated for 30 min and kept constant weight. The extract solution was filtered with an organic needle filter. Then, their contents were analyzed with an HPLC system featuring an Agilent HP1260 infinity with a UV VWD detector (Agilent, USA). The temperature of the selected XBridge SB C-18 column (5 μm, 4.6 mm × 250 mm) was 30 °C. Eluted with methanol/0.1% acetic acid-water (3:97) for 5 min, then with methanol/water (3:97–95:5) for 18 min. Flow rate was 0.8 mL/min, and detection wavelengths were 272 nm (caffeine), 268 nm (trigonelline), and 254 (chlorogenic acid), respectively.

### 2.3 The sensory analysis

Green coffee beans were subsequently roasted to a medium-roasted degree using a IKWA Pro V3 coffee bean roaster (IKAWA Ltd., London, United Kingdom) under 10–15 min roasting time, 220 °C roasting temperature to obtain brown roasted coffee beans. Then, the roasted coffee samples were distributed to three Q-grader coffee connoisseurs from Anke Coffee Co. Ltd. (Kunming, China) for sensory analysis using the Specialty Coffee Association (SCA) under the double-blinded contition. Firstly, the roasted coffee beans were ground into powder to evaluate the smell. Afterwards, the smell and taste were evaluated under the following brewing conditions: 1:18.18 (coffee powder: water, *w/w*) at 93 °C for 4 min, filtered residues, after 10 min. The aroma/fragrance, clean cup, aftertaste, sweetness, uniformity, balance, body, acidity, flavor, and overall impression were scored using a 10-point scale, while the body, aftertaste, aroma, and fragrance characteristics were described in detail. Finally, coffee with a final total score above 80 was regarded as specialty grade.

### 2.4 Microbial diversity assessment

Majorbio Bio-Pharm Technology Co. Ltd. in Shanghai (China), performed the Illumina amplicon sequencing of the coffee cherries, while a FastPure Stool DNA Isolation Kit (Magnetic Beads) from MJYH in Shanghai (China) was employed to extract the total microbial genomic DNA. The 16S rRNA genes were sequenced via PCR amplification using the bacterial 799F primer and 1193R reverse primer. The ITS3F and ITS4R primers were employed to amplify the fungal ITS region. The PCR amplification process included a 3-min denaturation at 95 °C, an additional 27 denaturation repetitions for 30 s each at 95 °C, 30-s annealing at 55 °C, a 45-s extension for at 72 °C, a 10-min extension at 72 °C, and termination at 4 °C. The second round was carried out in 13 cycles. A paired-end technique was employed to sequence the equimolar purified amplicon concentrations using a NextSeq 2000 system (Illumina, San Diego, United States). Then, the raw sequencing information was entered into the NCBI Sequence Read Archive (SRA) database (Accession Numbers: PRJNA1251849 and 1,251,855).

### 2.5 Non-volatile metabolite examination

The metabolites in the coffee cherries were assessed via UPLC-MS/MS. A 150 mg coffee sample and 1.20 mL of an 80% methanol–water mixture (*v/v*) with four different internal standard solutions (0.02 mg/mL L-2-chlorophenylalanine) were ground for 6 min at −10 °C, ultrasonically extracted for 30 min at 40 kHz and 5 °C and left to stand for 30 min at −20 °C, followed by a 15-min centrifugation at 4 °C and 13,000 g. The supernatant was analyzed via UPLC-MS/MS using a method described by Shen et al. (26). The UHPLC-Q Exactive HF-X system was equipped with an ACQUITY UPLC HSS T3 column (100 mm × 2.1 mm i.d. and 1.8 μm; Waters, United States). Furthermore, mobile phase A consisted of 0.1% formic acid in a acetonitrile-water mixture at a ratio of 5:95 (*v/v*) while mobile phase B comprised 0.1% formic acid in an acetonitrile-isopropanol-water solution at a 47.5:47.5:5 ratio (*v/v*). The other parameters included a 40 °C column temperature and a 3 μL injection volume. The gradient elution process consisted of 0 to 5% B from 0–0.1 min; 5 to 25% B from 0.1–2 min; 25 to 100% B from 2–9 min; 100% B from 9–13 min; and 100 to 0% B from 13–13.1 min; followed by 0% B from 13.1–16 min to balance the system. A UHPLC-Q Exactive HF-X MS system featuring an electrospray ionization source was employed in positive and negative ion modes. The mass spectrum parameters included a 400 °C heating temperature, 3,500 V and −3,500 V ion-spray voltages floating (ISVF) in positive and negative modes, respectively, a 20–40-60 V normalized collision energy for MS/MS, and a 70–1,050 m/z mass detection range. Finally, the pretreatment of raw data was performed by Progenesis QI (Waters Corporation, Milford, United States) software. A three-dimensional data matrix including sample information, metabolite name and mass spectral response intensity was exported. Then, internal standard peaks, as well as any known false positive peaks were removed from the data matrix, deredundant and peak pooled. Finally, the metabolites were identified by searching database, and the main databases were the HMDB,^1^ Metlin^2^ and the self-compiled Majorbio Database (MJDB) of Majorbio Biotechnology Co., Ltd. (Shanghai, China). At the same time, the data matrix obtained by searching the database was uploaded to the Majorbio cloud platform^3^ for data analysis. At least 80% of the metabolic features detected in any set of samples were retained. After filtering, the minimum value in the data matrix was selected to fill the missing value and each metabolic signature was normalized to the sum. The response intensities of the sample mass spectrometry peaks were normalized using the sum normalization method to obtain the normalized data matrix. Meanwhile, the variables of quality control (QC) samples with relative standard deviation (RSD) > 30% were excluded and log10 logarithmicized to obtain the final data matrix for subsequent analysis.

### 2.6 Statistical analysis

Each group was analyzed in triplicate (*n* = 3). The results were expressed as mean values with standard deviations (SD). One-way ANOVA was employed to determine signifcant differences (*p* ≤ 0.05) between the control and exposure groups. An Student–Newman-Keulsa comparison test was used to assess these differences (IBM SPSS Statistical 20.0). The R package “ropls” (Version 1.6.2) was used to perform orthogonal least partial squares discriminant analysis (OPLS-DA). Variables with values of *p* ≤ 0.05, VIP > 1.0, and a fold change (FC) > 1.5 or < 0.67 were categorized as differentially changed metabolites (DCMs).

## 3 Results

### 3.1 The contents of caffeine, trigonelline and chlorogenic acid

**Figure 1:**
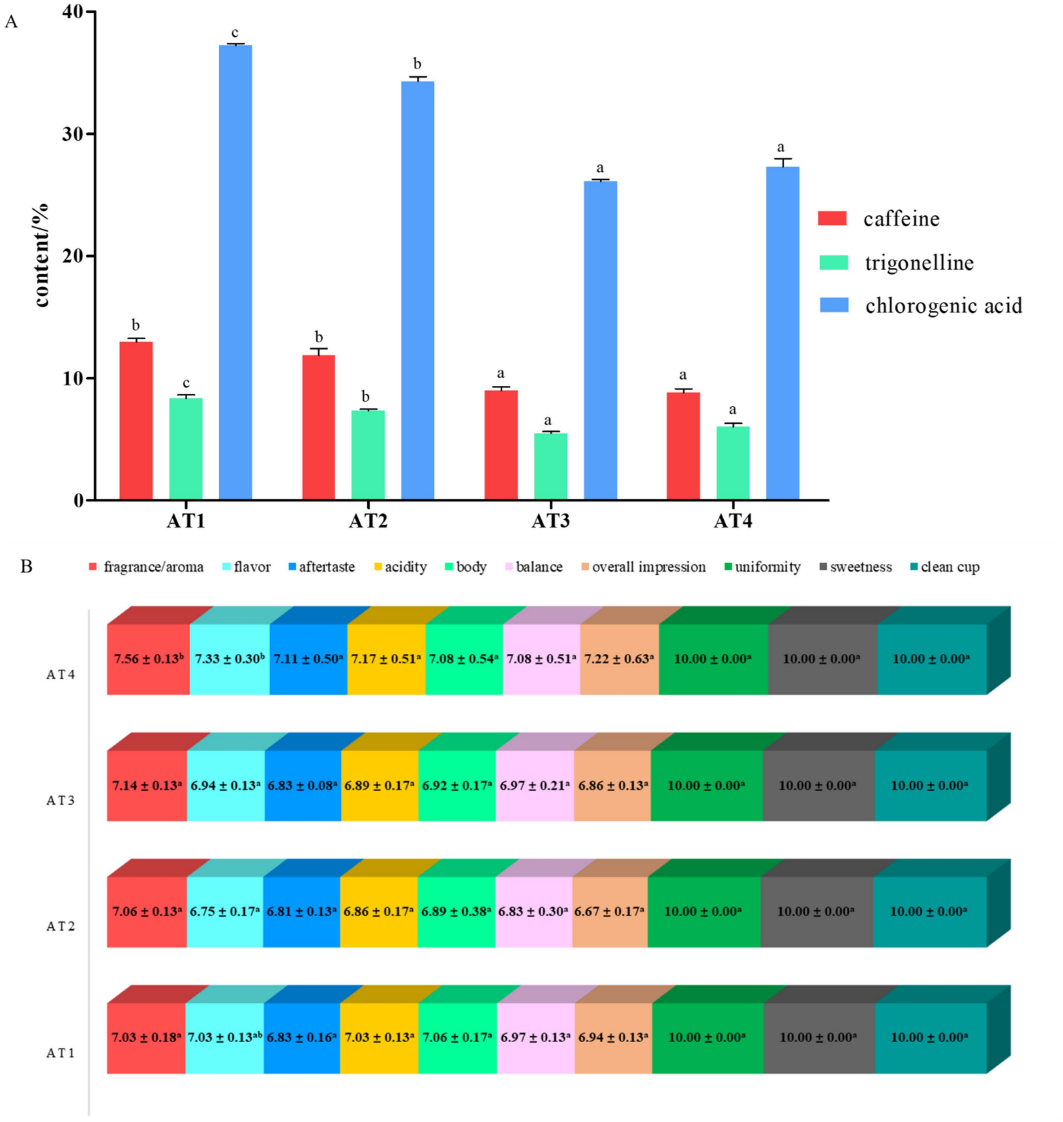
The contents of caffeine, trigonelline, and chlorogenic acid from different altidude coffee beans **(A)**, the sensory anaysis of different altitudes **(B)**.

The calibration curves, linearity range, limits of detection and quantification, validation parameters, and compound identification were shown in Table 1. Based on the calibration curves, the contents of caffeine, trigonelline, and chlorogenic acid were calculated (Figure 1A). The contents of caffeine, trigonelline and chlorogenic acid in low altitude coffee were significantly different from high altitude coffee, while they were not significantly different in 1400 m and 1,600 m. The coffee beans at 1000 m had the highest contents of caffeine, trigonelline and chlorogenic acid with (12.97 ± 0.51), (8.35 ± 0.50), and (37.24 ± 0.28)%, respectively.

**Table 1:** The calibration curves of of caffeine, trigonelline and chlorogenic acid.

Evaluation indicators of HPLC analysis method		Caffeine	Trigonelline	Chlorogenic acid
Calibration curves		*Y* = 12.1*X* + 10.1	*Y* = 16.2*X* – 3.67	*Y* = 38.3*X* + 17.7
Linearity range/(mg/100 g)		10–200	10–200	10–200
Limits of detection/(mg/100 g)		0.04	0.03	0.02
Quantification/(mg/100 g)		0.15	0.15	0.15
R^2^		0.999	0.999	0.999
Standard recovery rate/%	High content	90.57	93.77	98.31
	Middle content	95.70	102.90	87.77
	Low content	106.97	95.98	105.18

### 3.2 The results of sensory analysis

The scores of detailed attributes were evaluated as shown in Figure 1B. The score of uniformity, sweetness, and clean up with 10 meant these coffee samples were the absence of defect. The results of cupping quality showed that the cupping score of AT4 was the highest with 80.56 ± 2.89 in different altitude coffee samples, which meant coffee from AT4 was regarded as specialty grade. Fragrance/aroma and flavor of AT4 were significantly different with other altitude coffee. In addition, AT4 had strong fruitly characteristic, obvious roast cereal and nut aroma.

### 3.3 Microbial diversity evaluation

**Figure 2:**
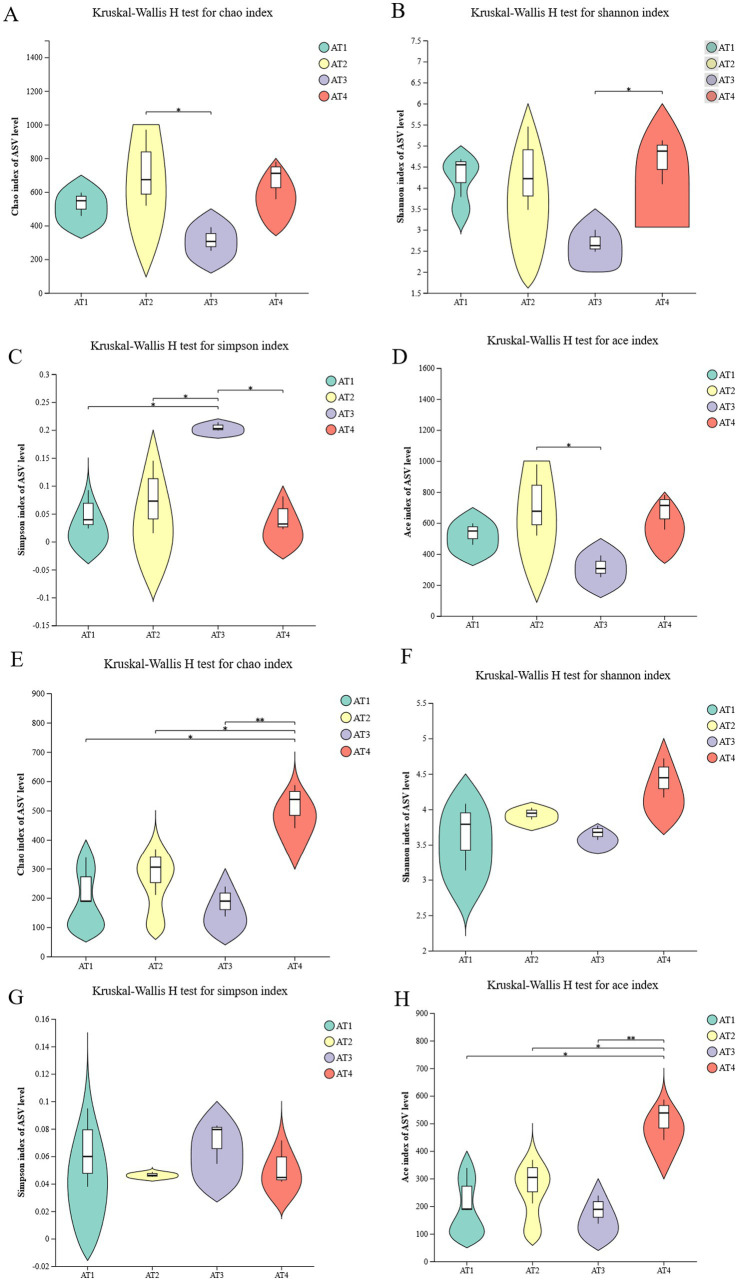
The microbial alpha diversity. **(A–D)** Chao, Shannon, Simpson, and ACE indices in bacteria, respectively; **(E–H)** Chao, Shannon, Simpson, and ACE indices in fungi, respectively.

The results showed an optimized total of 802,385 sequences for bacteria and 962,447 for fungi, with a collective total of 302,431,929 bases for bacteria and 294,154,598 for fungi. The average sequence lengths were 377 bp and 306 bp, respectively. The alpha diversity (Figure 2) was used to assess the microbial biodiversity. For bacteria, the Chao and ACE indices in AT2 were the highest, reaching 725.77 ± 255.10 and 729.58 ± 259.63, while the lowest values were 316.67 ± 78.55 and 316.75 ± 78.43 in AT3. The Simpson index ranged from 0.047 ± 0.035 (AT4) to 0.21 ± 0.0086 (AT3), whereas the Shannon index ranged from 2.71 ± 0.30 (AT3) to 4.67 ± 0.60 (AT4). For fungi, the AT4 exhibited the highest *α*-diversity (Chao, Shannon, and ACE index), reaching 519.81 ± 93.22, 4.44 ± 0.31, and 519.62 ± 82.97, respectively. The Simpson index ranged from 0.047 ± 0.0020 (AT2) to 0.071 ± 0.017 (AT3). The beta diversity was analyzed using Mothur (version 1.30.2) to obtain principal component analysis (PCA), non-metric multidimensional scaling (nMDS), and permutational multivariate analysis of variance (PERMANOVA; Supplementary Figure S1). PCA with PC1 (37.25%) and PC2 (19.67%) in bacteria showed the discrimination of the four different altitude coffee samples, which explained 56.92% of variance. In fungi, PC1 (29.84%) and PC2 (16.17%) explained 46.01% of variance. The nMDS analysis showed the distribution of the four different altitude samples, highlighting differences in the microbial community profiles. Among the fungal communities, the most remarkable dissimilarity was observed at four planting altitudes. Within the bacterial communities, AT3 and AT4 exhibited the greatest dissimilarity. Moreover, the PERMANOVA showed R^2^ is 0.47 (*p* = 0.009) in bacteria, 0.49 (*p* = 0.001) in fungi, respectively.

**Figure 3:**
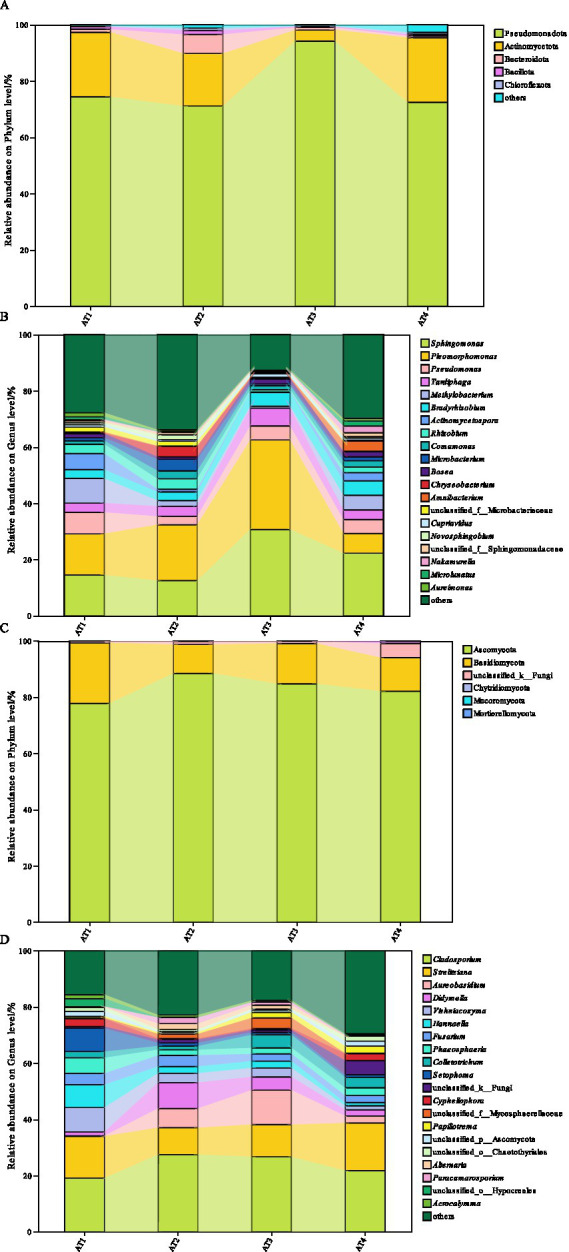
The relative abundance percentages of different planting altitudes, at the phylum level of bacteria **(A)**, the genus level of bacteria **(B)**, the phylum level of fungi **(C)**, and the genus level of fungi **(D)**.

Six bacterial phyla, including *Pseudomonadota*, *Actinomycetota*, *Bacteroidota*, *Bacillota*, *Chloroflexota*, and others, were identified from four planting altitudes of coffee (Figure 3A). Among them, *Pseudomonadota* was the dominant bacterial phylum, which ranged from 71.17% (AT2) to 94.21% (AT3), reaching the maximum relative abundance at 1400 m. After *Pseudomonadota*, *Actinomycetota* was also an important bacterial phylum, which ranged from 3.97% (AT3) to 23.02% (AT4). In AT2, *Bacteroidota* occupied 6.76%. 21 genera, including *Sphingomonas*, *Pleomorphomonas*, *Pseudomonas*, *Tardiphaga*, *Methylobacterium*, *Bradyrhizobium*, *Actinomycetospora*, *Rhizobium*, *Comamonas*, *Microbacterium, Bosea*, *Chryseobacterium*, *Amnibacterium*, *Cupriavidus*, *Novosphingobium*, *Nakamurella*, *Microlunatus*, *Aureinonas*, and others were identified (Figure 3B). According to the relative abundance on the genus level, *Sphingomonas* (12.52% ~ 30.74%) and *Pleomorphomonas* (7.00% ~ 31.82%) were the dominant bacterial genera in coffee from different planting altitudes. Meanwhile, *Sphingomonas* and *Pleomorphomonas* showed the maximum relative abundance in AT3.

For fungi, six phyla (*Ascomycota*, *Basidiomycota*, *Chytridiomycota*, *Mucoromycota*, unclassified fungi, and others) were identified at the phylum level (Figure 3C). *Ascomycota* was the dominant fungal phylum, which ranged from 77.84 to 88.52%. The maximum relative abundance was 88.52% in AT2. After *Ascomycota*, *Basidiomycota* was also an important fungal phylum from 10.30% (AT2) to 21.54% (AT1). 21 genera were identified, including *Cladosporium*, *Strelitziana*, *Aureobasidium*, *Didymella*, *Vishniacozyma*, *Hannaella*, *Fusarium*, *Phaeosphaeria*, *Colletotrichum*, *Setophoma*, *Cyphellophora*, *Papiliotrema*, *Alternaria*, *Paracamarosporium*, *Acrocalymma*, and others (Figure 3D). The *Cladosporium* and *Strelitziana* fungi showed the highest relative abundance at the genus level, ranging from 19.11% (AT1) to 27.49% (AT2) and 9.57% (AT2) to 16.94% (AT4), respectively. In addition, *Vishniacozyma* (8.69%) and *Setophoma* (8.27%) were also important in AT1. *Setophoma* and *Didymella* occupied 9.57 and 9.24% in AT2, 11.46 and 4.67% in AT3, respectively. However, *Colletotrichum* occupied 5.04% in AT4.

### 3.4 The results of UPLC-MS/MS analysis

**Figure 4:**
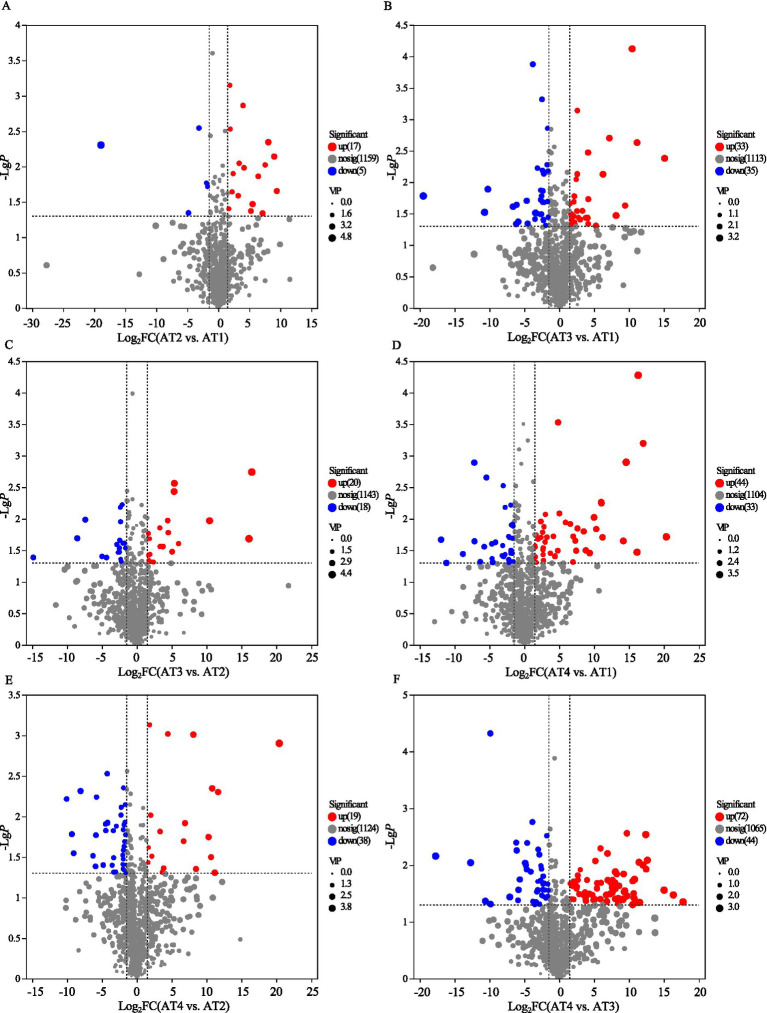
The differentially changed non-volatile compounds (DCMs) between AT2 vs. AT1 **(A)**, AT3 vs. AT1 **(B)**, AT3 vs. AT2 **(C)**, AT4 vs. AT1 **(D)**, AT4 vs. AT2 **(E)**, and AT4 vs. AT3 **(F)**, respectively.

A total of 1,040 metabolites across 16 super-classes were identified in the coffee from different planting altitudes (Supplementary Figure S2). Furthermore, the differentially changed metabolites (DCMs) of the coffee from different planting altitudes with VIP > 1.0, *p ≤* 0.05, and FC < 0.67 or VIP > 1.0, *p ≤* 0.05, and FC > 1.5 were shown in Figure 4. Of the 22 DCMs detected in the AT2 and AT1 comparison, 17 showed up-regulation (e.g., cycloastragenol, avocadene, leukotriene D5, tsugarioside B, etimicin, pantothenic acid, tetracycline, tricaffeoyl spermidine, and zerano, desacetylvinblastine amide, diisopropanolamine, palmitoyl Ara-C, methionyl-tyrosine, etc.) and five exhibited down-regulation (e.g., stevioside, 7Alpha-hydroxytestosterone, isopimaric acid, fusarin C, thapsigargin; Figure 4A).

Similarly, 68 DCMs identified in the AT3 and AT1 comparison, 33 showed up-regulation (e.g., cycloastragenol, secoisolariciresinol, D-sorbitol, Lac Dye, sambacin, rosolic acid, trifarotenethapsigargin, vignatic acid A, spirolide C, pinostrobin 5-glucoside and licoflavanone, ssecoisolariciresinol, etc.) and 35 exhibited down-regulation (e.g., cephaloglycin, fucoxanthin, Ac-(5H-dibenzyl(A, D)cycloheptene-10,11-dihydroglycine-Leu-Asp-Ile-Ile-Trp), quercetin 3-sophorotrioside 7-rhamnoside, doxorubicinol, and fevicordin B 2-[rhamnosyl-(1- > 4)-glucosyl-(1- > 6)-glucoside], 2,3-butanediol apiosylglucoside, nitrobenzyl-6-thioinosine, etc.; Figure 4B).

Of the 38 DCMs detected in the AT3 and AT2 comparison, 20 showed up-regulation (e.g., stevioside, (3B,20R,22R)-3,20,27-trihydroxy-1-oxowitha-5,24-dienolide 3-glucoside, (5Alpha,6Beta,14Alpha,20R,22R)-5,6,14,20,27-pentahydroxy-1-oxowith-24-enolide, gambogic acid, etc.) and 18 exhibited down-regulation (e.g., gamabufotalin, (S)-succinyldihydrolipoamide, alliofuroside A, sitosterol Beta-D-glucoside, Ile-Ile-Ile-Pro, arginyl-arginyl-arolyl-tyrosyl-isoleucyl-leucine, dynorphin A 1–8, microcystin-Lr, notoginsenoside J, tricaffeoyl spermidine, Ac-(5H-dibenzyl(A, D)cycloheptene-10,11-dihydroglycine-Leu-Asp-Ile-Ile-Trp), etc.; Figure 4C).

Of the 77 DCMs identified in the AT4 and AT1 comparison, 44 were up-regulated (e.g., 5-butyl-3,4-dimethyl-2-furanundecanoic acid, glucosylgalactosyl hydroxylysine, tricaffeoyl spermidine, epothilone A, camelliaside B, 5-deoxykievitone hydrate, erythromycin, davercin, des-arg(9)-bradykinin, ebiratide, leucine-betaxanthin, tyrosyl-phenylalanine, etc.) and 33 were down-regulated (e.g., stigmasterol, 28-glucopyranosyl-3-methyloleanolic acid, 5-hydroperoxyeicosatetraenoic acid, methionyl-tyrosine, isopenicillin N, and lyciumin B, cyromazine, 3-carboxy-1-hydroxypropylthiamine diphosphate, 4-pyridoxic acid, 5-heptyltetrahydro-2-oxo-3-furancarboxylic acid, triethylene glycol bis(3-tert-butyl-4-hydroxy-5-methylphenyl)propionate, etc.; Figure 4D).

Of the 57 DCMs detected in the AT4 and AT2 comparison, 19 were up-regulated (e.g., tetranorprostanedioic acid, 5-hydroperoxyeicosatetraenoic acid, 3-carboxy-1-hydroxypropylthiamine diphosphate, succinic acid, methionyl-tyrosine, D-2-hydroxyglutaric acid, lyciumin B, D-erythrose, temazepam glucuronide), (4-(2-(3-(cyclopentyloxy)-4-methoxyphenyl)-2-phenylethyl)pyridine, etc.) and 38 were down-regulated (e.g., 10-nitrooleate, clofibryl glucuronide, gambogic acid, etc.; Figure 4E).

Of the 116 DCMs detected in the AT4 and AT3 comparison, 72 showed up-regulation (e.g., calyculin A, araloside A, kirenol, cyclosquamosin G, cyclosquamosin C, tyrosyl-phenylalanine, clofibryl glucuronide, cryptochlorogenic acid, melezitose, sucrose, Alpha-solanine, 4-oxo-enoxacin, desacetylvinblastine amide, 3-(3,5-dichlorophenyl)-4-hydroxy-1,3-thiazol-2-one, nitrobenzyl-6-thioinosine, etc.) and 44 exhibited down-regulation (e.g., (9Z)–(13S)-12,13-epoxyoctadeca-9,11-dienoate, 27-hydroxyisomangiferolic acid, glutamylleucylarginine, succinic acid, lyciumin B, D-2-hydroxyglutaric acid, fructose-6-phosphate, 4-[(2-furanylmethyl)thio]-2-pentanone, 4-pyridoxic acid, tricin 7-[rhamnosyl-(1- > 2)-galacturonide], guanosine 5′-monophosphate, etc.; Figure 4F).

**Figure 5:**
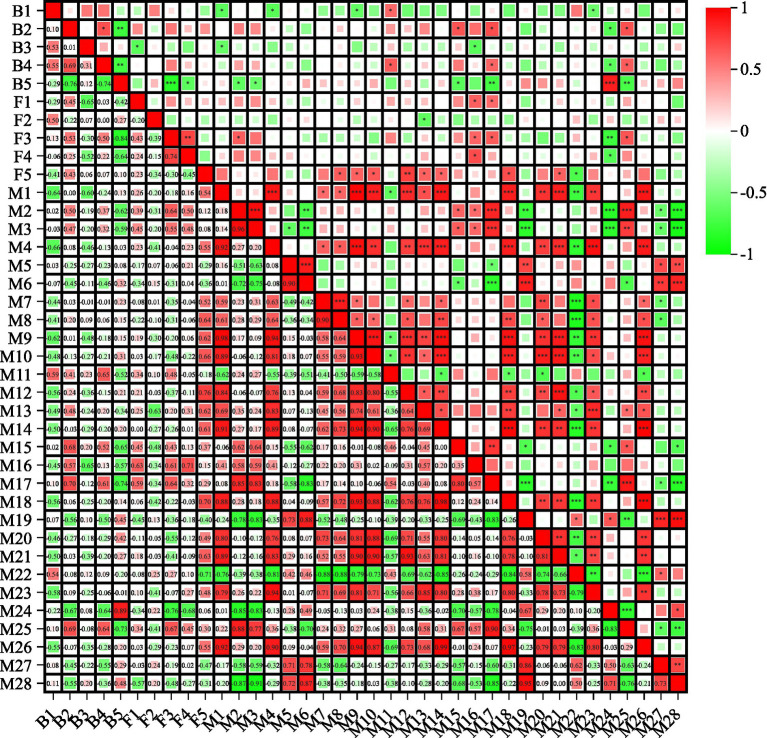
The differentially changed metabolites (DCMs) between four altitudes samples. B1: *Sphinomonas*, B2: *Pleomorphomanas*, B3: *Pseudomonas*, B4: *Tardiphaga*, B5: *Methylobacterium*, F1: *Cladosporium*, F2: *Strelitziana*, F3: *Aureobasidium*, F4: *Didymella*, F5: *Vishniacozyma*, M1: (S)-bromoenol lactone, M2: 27-hydroxyisomangiferolic acid, M3: 3-epicorosolic acid, M4: 6”-O-acetyldaidzin, M5: alliofuroside A, M6: alpha-solamarine, M7: Arg-Ala-Ile, M8: bullatanocin, M9: camellianin A, M10: cephaloglycin, M11: chrysoobtusin, M12: corilagin, M13: D-2-hydroxyglutaric acid, M14: dihydro-2-methoxy-2-methyl-3(2H)-thiophenone, M15: echinocystic acid-3-O-glucoside, M16: fistulosin, M17: fructose-6-phosphate, M18: gamma-butyrolactone, M19: hoduloside IV, M20: kirenol, M21: lactose citrate, M22: notoginsenoside E, M23: oxazepam glucuronide, M24: phenylalanine-betaxanthin, M25: sumaresinolic acid, M26: thienodiazepine, M27: tricaffeoyl spermidine, M28: vinleucinol.

Finally, the VIP in the OPLS-DA model (R^2^ = 0.904, Q^2^ = 0.826) and the *p* value of the one-way ANOVA were used to identify DCMs between different altitude samples with a threshold of VIP > 1 and *p* ≤ 0.05, 28 DCMs were found (Table 2). Moreover, the correlation between these DCMs and bacteria, fungi with the top five relative abundances was analyzed (Figure 5). Based on the value of the correlation coefficient (*r*), 0.8 ~ 1.0 indicated an extremely strong correlation, 0.6 ~ 0.8 indicated a strong correlation (27). *Sphinomonas* was strongly negatively correlated with (S)-bromoenol lactone, M4 and camellianin A. *Pleomorphomanas* was strongly negatively correlated with phenylalanine-betaxanthin, and was strongly positively correlated with echinocystic acid-3-O-glucoside, fructose-6-phosphate, and sumaresinolic acid. *Pseudomonas* was strongly negatively correlated with (S)-bromoenol lactone and fistulosin. *Tardiphaga* was strongly negatively correlated with phenylalanine-betaxanthin, and was strongly positively correlated with (S)-bromoenol lactone1, fructose-6-phosphate, and sumaresinolic acid. *Methylobacterium* was strongly negatively correlated with 27-hydroxyisomangiferolic acid, echinocystic acid-3-*O*-glucoside, fructose-6-phosphate, sumaresinolic acid, and was strongly positively correlated with phenylalanine-betaxanthin. *Cladosporium* was strongly positively correlated with M6. *Strelitziana* was strongly negatively correlated with D-2-hydroxyglutaric acid. *Aureobasidium* was strongly negatively correlated with phenylalanine-betaxanthin, and was strongly positively correlated with 27-hydroxyisomangiferolic acid, fistulosin, fructose-6-phosphate and sumaresinolic acid. *Didymella* was strongly negatively correlated with phenylalanine-betaxanthin, and was strongly positively correlated with fistulosin. *Vishniacozyma* was strongly negatively correlated with notoginsenoside E, and was strongly positively correlated with bullatanocin, camellianin A, cephaloglycin, corilagin, D-2-hydroxyglutaric acid, dihydro-2-methoxy-2-methyl-3(2H)-thiophenone, gamma-butyrolactone and lactose citrate.

**Table 2:** Differentially changed metabolites (DCMs) between samples (VIP > 1.0 and *p* ≤ 0.05).

No.	Metabolite	VIP	*p* value
M1	(S)-bromoenol lactone	1.5926	0.0396
M2	27-hydroxyisomangiferolic acid	1.3800	0.0273
M3	3-epicorosolic acid	1.4759	0.0237
M4	6”-O-acetyldaidzin	1.4928	0.0329
M5	alliofuroside A	1.5979	0.0345
M6	alpha-solamarine	2.1332	0.0378
M7	Arg-Ala-Ile	1.6012	0.0415
M8	bullatanocin	1.4553	0.0307
M9	camellianin A	1.6730	0.0396
M10	cephaloglycin	1.7073	0.0378
M11	chrysoobtusin	1.0423	0.0415
M12	corilagin	1.4589	0.0345
M13	D-2-hydroxyglutaric acid	1.326	0.0329
M14	dihydro-2-methoxy-2-methyl-3(2H)-thiophenone	1.1039	0.0361
M15	echinocystic acid-3-O-glucoside	1.5868	0.0455
M16	fistulosin	1.3845	0.0499
M17	fructose-6-phosphate	1.0697	0.0329
M18	gamma-butyrolactone	1.3929	0.0329
M19	hoduloside IV	1.2028	0.0329
M20	kirenol	2.7825	0.0261
M21	lactose citrate	1.4623	0.0329
M22	notoginsenoside E	2.6189	0.0216
M23	oxazepam glucuronide	1.1148	0.0286
M24	phenylalanine-betaxanthin	1.1850	0.0216
M25	sumaresinolic acid	1.3164	0.0445
M26	thienodiazepine	1.1051	0.0378
M27	tricaffeoyl spermidine	1.4927	0.0476
M28	vinleucinol	1.5916	0.0329

## 4 Discussion

Coffee flavor is the most crucial competitive condition in the coffee market. Previous studies have verified that many factors, such as coffee species, geographical origin, climatic conditions, cultivation practices, post-harvest primary processing methods, storage conditions, degree of roasting, and organoleptic qualities, can influence coffee quality and coffee flavor (23, 28). Among these factors, planting altitude contributes 74.2% to coffee flavor characteristics based on the Random Forest model score (29). This may be caused by the following reasons. One originates from the plant-associated microbiomes under local conditions. Microorganisms play a key role in the coffee industry. One main reason is that rich microorganisms coming from coffee cherries, soil, air, water, etc., produce enzymes to degrade pectin and produce metabolites to change chemical compounds (26, 30). However, these microorganisms are easy effected by coffee maturation, primary processing, and other factors during coffee planting and processing (31, 32). Among them, planting altitudes can also cause significant differences in the microbial richness and dominant microorganisms (18). Based on the function of microbiota, plant-associated microorganisms and local environmental conditions, farm management factors, and plant characteristics are interactional relationship (33). The planting microbiome is influenced by regional environmental factors, which create unique conditions for the coffee microbial terroir, encompassing various elements, including soil composition, agricultural practices, interactions with other organisms, topography, landscape, and climate. Therefore, the microbiota in coffee are collectively impacted by processing techniques, genetic aspects, and environmental conditions. The microbiome does not exist in isolation, which actively influences the performance of the plant and the sensory properties of the crop (34). Specifically, the microbiome associated with coffee cherries may contribute significantly to the flavor profile of the final product. This occurs through the generation or modification of the volatile substances in the fruit during the growth period or by playing a key role in the first fermentation stages after harvesting (34). Because the microbial fermentation of coffee usually involves the direct use of environmental microbes of the coffee plant and endophytic microorganisms in coffee. Variations in the microbiomes of different plant organs can cause phytochemical changes by modifying the metabolism of the host. For instance, this can enhance nutrient uptake by the roots, followed by distribution to the fruits, seeds, and leaves. The microbes in plants can consume molecules, removing them from the metabolome, or they can contribute smells and flavors of their own (34). Therefore, plant-associated microbiomes from coffee have emerged as a potential driver of coffee flavor. This insight can help predict the flavor profiles of coffee and steer future initiatives aimed at enhancing farms for specialty quality properties (33). The other is related to the function of microbiomes in coffee fermentation during post-harvest. Microbiomes from various sources, such as people, equipment, air, and soil, form a diverse, active that significantly influences the quality of coffee via fermentation (30, 35). Varied microbiota has been identified in both the coffee cherries and the surrounding soil. Since microbial proliferation during fermentation can impact product quality, it is crucial to consider how production areas, coffee cultivars, and processing techniques interact with the microbiota in coffee. This allows for the selection of optimal starter cultures or fermentation conditions, aimed at enhancing specific terroir properties or adjusting the microbial community to promote the introduction of advantageous microorganisms to improve the quality of coffee (36).

This paper used the alpha diversity to reflect microbial community richness and microbial community diversity of different planting altitudes (37, 38). The Chao index suggested that coffee at 1200 m (AT2) presented the highest bacterial richness, 1,600 m (AT4) had the highest fungal richness. The Shannon index suggested that coffee at 1400 m (AT3) had the greatest complexity. Moreover, *Sphingomonas* and *Pleomorphomonas*, the dominant bacterial genera, were significantly different in different planting altitudes with the maximum relative abundance in AT3. *Sphingomonas*, a Gram negative bacterium, is widely distributed and originate from glaciers, plants, water, air, reservoirs, soil, freshwater sediments, etc. (39). *Sphingomonas* can promote plant growth and enhance stree resistance by secreting hormones or attracting beneficial bacterial communities (39). *Sphingomonas* also can be used for food industry, oil collecting industry, construction industry based on the ability of secreteing sphingan (39). *Pleomorphomonas* are highly adaptable to low temperatures, which can contribute to fermentation and support substrate turnover (40). For fungi, *Cladosporium* and *Strelitziana* were the dominant fungal genera while *Vishniacozyma* and *Setophoma* were also important in AT1. *Setophoma* and *Didymella* in AT3. These results are consistent with the study by Tapaça, who pointed out planting altitude shapes the dynamics of microbial communities (41). This study also showed the abundance and richness of soil microorganisms furtherly influence microbial biomass and moisture and different metabolic adaptations by different altitudes (41). *Cladosporium* is often commonly found in plants, fungi, soil, air, and other organic debris, which also are beneficial to plants by promoting plant growth or enhancing plant resistance to biotic and abiotic stress (42, 43). These results reflected that the influence of planting altitude on the microbial community structure may influence coffee growth and coffee fermentation.

In addition, coffee species, geographical origin, climatic conditions, cultivation practices, and other vital factors also can influence coffee flavor precursor substances, which can format flavor compounds (such as lipids, polysaccharides, sucrose, proteins, chlorogenic acid, caffeine, and trigonelline) by Maillard reactions, Strecker degradation, caramelization, and thermal fragmentation to produce aroma-active compounds during roastion (44). Under the interaction of these factors, the formation and accumulation of these coffee flavor precursor substances undergo differential accumulation in green coffee beans. Research has shown that in areas below 969 m, the key substances contributing to the distinctive chemical profile of coffee were malate, citrate, trigonelline, and lipids. Contrarily, at elevations above 1,000 m, formic acid, caffeine, and quinic acid became the primary contributors (45). Simultaneously, according to the analysis results of non-volatile compounds, some significant differences in compounds were found. These DCMs are attributed to the combined effect of different microbial fermentations and endogenous chemical compounds from different planting altitudes. The content of caffeine, trigonelline and chlorogenic acid fluctuated with increasing altitudes, which would vary coffee sernory. Because caffeine, trigonelline, and chlorogenic acid contribute to the flavor, aroma or bitter taste attributes of coffee (46, 47). Caffeine influences the perceived strength, body, and bitterness of coffee, while trigonelline contributes to the overall aroma and positively correlates with coffee astringency and aftertaste astringency (25, 48). According to the analysis of caffeine, trigonelline and chlorogenic acid content, high altitude coffee had lower caffeine and trigonelline contents. Chlorogenic acids can contribute to the astringency and bitterness of coffee (49). High-altitude coffee had reduced caffeine and chlorogenic acid contents, which were consistent with the results reported by Hu et al. (3). At the same time, the sensory analysis showed hight-altitude coffee had a higher cupping score and better aroma and flavor than low altitude coffee. Therefore, planting altitude enhance aroma and flavor because high-altitudes can affect the chemical composition of coffee cherries and beans both prior and after harvested (18, 29). Endogenous chemical compounds of coffee seeds and microbial fermentation originating are affected by planting altitudes. Overall, the planting altitude will contributes to coffee flavor by affecting the microbiome characteristics and endogenous chemical compounds. This suggests that efforts aimed at enhancing microbiomes for better quality must be customized to the unique systems and conditions of each farm.

China is a producer of specialty coffee that meets international standards. Yunnan Province is the most important coffee production region in China, which accounts for approximately 98% of China’s total coffee production. Moreover, Arabica coffee from Yunnan enjoys a high reputation in the international market for its unique flavor and high quality based on the unique high-altitude terroir, which contributes to the production of high-quality Arabica beans with distinctive flavor signatures. Specifically, Baoshan City, along the Gaoligong Mountains of Yunnan province, is one of the major coffee production areas in Yunnan and was known for producing specialty coffee (50). However, the study showed coffee from different regions in Yunnan also showed different flavor characteristics (24). So, one of the main weaknesses of this study is that different altitude coffee samples were only collected from Baoshan City. Puer City, Lincang City, and other main coffee production areas in China is not analyzed. More comprehensive coffee samples from different regions are not available. Therefore, planting altitude does not completely explain coffee flavor characteristics from different coffee regions. On the other hand, further sample information related to altitude, such as temperature, humidity, solar radiation, and soil properties, is difficult to accurately obtain in the study. This information also influences coffee quality.

In recent years, coffee fermentation using specific microorganisms has become a popular method to enhance coffee quality, resulting in an exceptional coffee brew (51, 52). So, microorganisms from high-altitude coffee will be potential starters in fermentation, which may be an effective measure to improve coffee flavor. Overall, the study on the microorganisms and chemical compounds of coffee from different altitudes will provide a reference for further study of coffee flavor formation and supple curreny research on coffee flavor. And it will have clear implications for coffee process strategies and product differentiation in coffee production systems.

Based on the observed correlations between microbial taxa and differentially changed metabolites (DCMs) across planting altitudes, we propose the following testable hypotheses for future validation studies. Altitude-driven shifts in the bacterial community composition (e.g., increased relative abundance of *Sphingomonas* and *Pleomorphomonas* at 1400 m) directly contribute to the observed decrease in caffeine, trigonelline, and chlorogenic acid contents at higher altitudes. A reciprocal transplant experiment: moving soil or cherry-associated microbiota from high altitude (1,600 m) to low altitude (1,000 m) and vice versa combined with long-term monitoring of flavor precursor compounds would allow causal inference. The fungal genera *Cladosporium* and *Strelitziana* positively regulate the synthesis or degradation of alpha-solamarine and D-2-hydroxyglutaric acid, respectively. This can be validated through co-culture experiments of coffee endophytes with these fungi, followed by metabolomic profiling and gene expression analysis of key biosynthetic enzymes. Testing these hypotheses will not only strengthen the causal understanding of microbe-metabolite interactions in coffee terroir but also provide actionable strategies for altitude-mimicking fermentation and quality improvement in the coffee industry.

## 5 Conclusion

The planting altitude is a critical factor influencing coffee flavor. To understand the influence of planting altitude on coffee cherries, this paper assessed microbial diversity and metabolites in arabica coffee between different planting altitudes in Yunnan province, China were analyzed. The dominant bacterial genera, *Sphingomonas* and *Pleomorphomonas*, were significantly different in different planting altitudes, with the maximum relative abundance in AT3. *Cladosporium* and *Strelitziana* were the dominant fungal genera, while *Vishniacozyma* and *Setophoma* were also important in AT1. *Setophoma* and *Didymella* in AT3. Meanwhile, the metabolites also significantly changed with the planting altitude, especially the important flavor precursor compounds (caffeine, trigonelline and chlorogenic acid). Therefore, the characteristics of microbiomes and endogenous metabolites of coffee are influenced by planting altitude, which will provide a affecting possibility of planting altitude on coffee quality.

## Data Availability

The raw data generated in this study have been submitted to the NCBI Sequence Read Archive (SRA) under accession numbers PRJNA1251849 and PRJNA1251855.

## Footnotes

1
http://www.hmdb.ca/

2
https://metlin.scripps.edu/

3
https://cloud.majorbio.com
