# Supplementary material for: Cherry microbiota and metabolites with planting altitude of Coffea arabica in Baoshan of China
Source: Front Nutr. 2026 May 4;13:1817512. doi: 10.3389/fnut.2026.1817512 (PMC13180847; doi:10.3389/fnut.2026.1817512)
Supplement: Supplementary file 1 [file Supplementary_file_1.docx]

**Figure S1.** The microbial β diversity (A: PcoA in bacteria; B: nMDS in bacteria; C: PcoA in fungi; D: nMDS in fungi).

**Figure S2.** Super-classes of chemical compounds of coffee from different planting altitudes, the different colors represented different super-classes.
